# Application of micro/nanorobot in medicine

**DOI:** 10.3389/fbioe.2024.1347312

**Published:** 2024-01-25

**Authors:** Tianhao Sun, Jingyu Chen, Jiayang Zhang, Zhihong Zhao, Yiming Zhao, Jingxue Sun, Hao Chang

**Affiliations:** ^1^ Department of Thoracic Surgery, The First Affiliated Hospital of Harbin Medical University, Harbin, Heilongjiang, China; ^2^ Department of Oncology, The Fourth Affiliated Hospital of Harbin Medical University, Harbin, Heilongjiang, China; ^3^ Key Laboratory of Carcinogenesis and Translational Research (Ministry of Education/Beijing), Department of Breast Oncology, Peking University Cancer Hospital and Institute, Beijing, China; ^4^ Department of Endocrinology and Metabolism, The Second Affiliated Hospital of Harbin Medical University, Harbin, China

**Keywords:** micro/nanorobot, biosensor, diagnosis, targeting drug delivery, surgery

## Abstract

The development of micro/nanorobots and their application in medical treatment holds the promise of revolutionizing disease diagnosis and treatment. In comparison to conventional diagnostic and treatment methods, micro/nanorobots exhibit immense potential due to their small size and the ability to penetrate deep tissues. However, the transition of this technology from the laboratory to clinical applications presents significant challenges. This paper provides a comprehensive review of the research progress in micro/nanorobotics, encompassing biosensors, diagnostics, targeted drug delivery, and minimally invasive surgery. It also addresses the key issues and challenges facing this technology. The fusion of micro/nanorobots with medical treatments is poised to have a profound impact on the future of medicine.

## 1 Introduction

Micro/nano robots refer to robots or robotic systems whose size is on the micron or nanometer scale. These robots typically consist of micro or nano-scale components and can perform specific tasks, such as operations at the cellular level or assembly and repair at the molecular level (Xie et al., 2019). With the rapid advancement of nanotechnology and materials science, micro/nano robots find applications in various fields of biomedicine (Mohammadinejad et al., 2016). The miniaturization of robot systems to the micro/nano scale holds immense potential in the diagnosis and treatment of various diseases (Nelson et al., 2010). These diminutive robots can penetrate deep or otherwise inaccessible regions within our bodies, conducting various medical procedures, and showcasing great promise in areas such as diagnosis, drug delivery, and surgery (Soto et al., 2020; Zhao et al., 2022).

Various methods exist for constructing micro/nano robots. Initially, one needs to define the tasks the micro/nano robot is intended to perform, specifying parameters such as size, shape, materials, sensors, actuators, and more. Physical methods like photolithography, chemical methods involving the synthesis of molecular components using chemical substances, or 3D printing technology can be employed to create parts. In addition, self-assembly technology enables the automatic assembly of nanoparticles into desired structures by controlling molecular interactions. When the size of an object is reduced to the micrometer or nanometer range, the ratio of inertial force to viscous force (Reynolds number) becomes negligible, making it necessary to continuously provide power to propel small robots. The driving modes for micro/nano robots primarily include chemical propulsion driven by local chemical and biochemical energy sources (e.g., H2O2, urea), physical field propulsion driven by external fields (e.g., light, ultrasound, or magnetic fields), and biological propulsion driven by microorganisms or cells (e.g., sperm). Building upon these propulsion methods, micro/nano robots can navigate deep or inaccessible regions of the body, performing various medical tasks and demonstrating significant potential in areas such as diagnosis, drug delivery, and surgery. For instance, in the realm of diagnostics, novel micro/nano machines are used for real-time measurements of blood glucose and lipid levels (Yu et al., 2021). These microdimmers are controlled by an applied magnetic field and exhibit distinct movement speeds and postures in response to different concentrations of glucose, cholesterol, and triglycerides, facilitating the measurement of blood glucose and lipids. In the field of surgery, micro/nano robots can access deep tissues that are beyond the reach of larger robots and human surgeons (Nguyen et al., 2021). For example, guided spiral robots have been employed for targeted thrombectomy and recanalization in small blood vessels (Nguyen et al., 2021). In the medical domain, micro/nano robots can accomplish tasks that are unattainable on a macro scale, thus enhancing the precision and refinement of medical treatments. This paper will primarily present research findings on the medical applications of micro/nano robots and elaborate on their current status and challenges in four key areas: biosensors, diagnostics, targeted drug delivery, and surgery.

## 2 Application of micro/nanorobot in medicine

### 2.1 Biosensor

**FIGURE 1:**
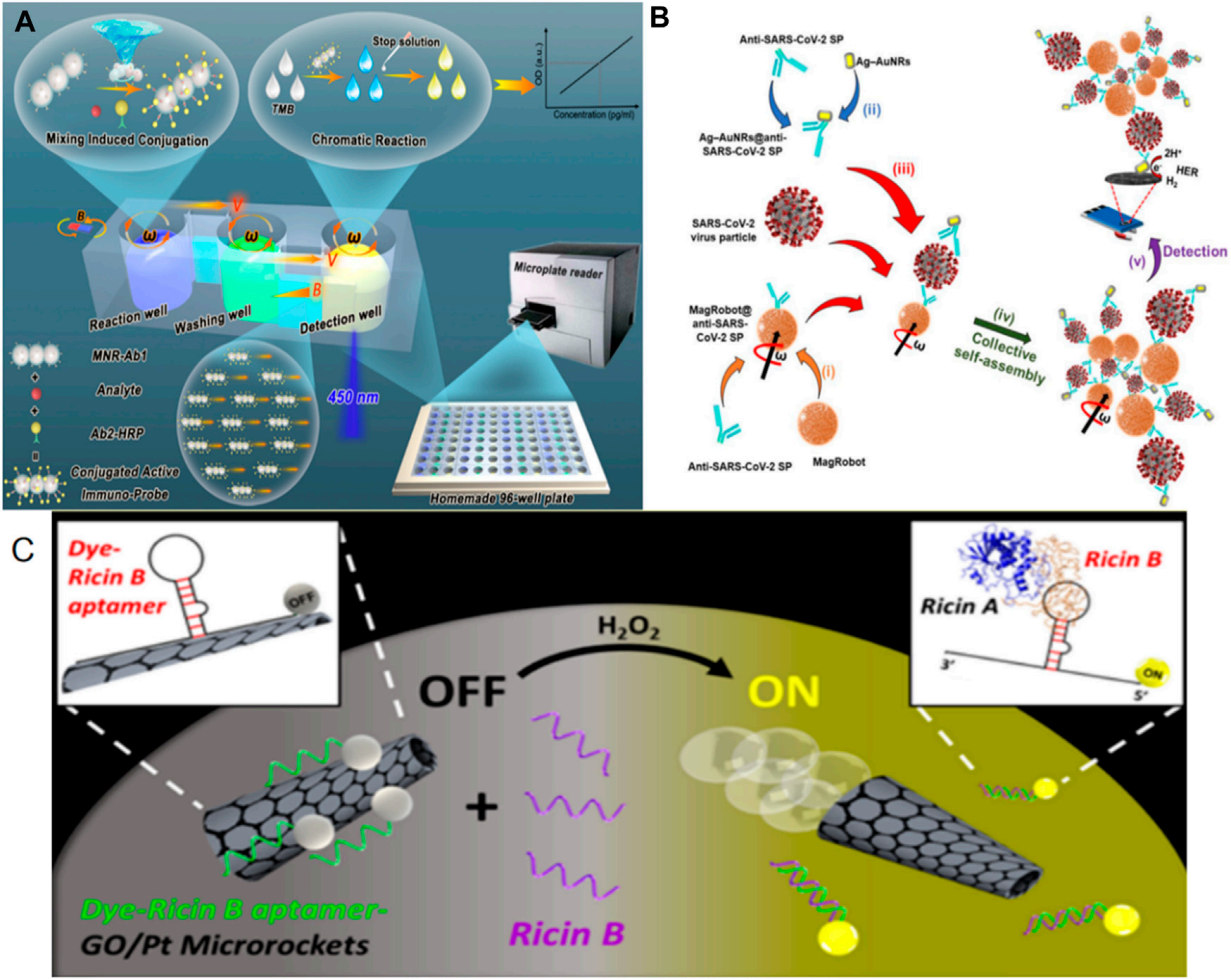
**(A)** Magnetic Nanorobot-Enabled Automated and Efficient ELISA (nR-ELISA) Analysis. Adapted with permission from Wang et al. (2022). Copyright 2022 American Chemical Society. **(B)** MagRobots were modified with antibody against SARS-CoV-2 spike protein. Adapted with permission from Mayorga-Martinez et al. (2022). Copyright 2022 Elsevier Science and Technology Journals. **(C)** The preparation of fluorescent magnetic spore–based (spore@Fe3O4@CDs) microrobots involves the combination of facile chemical deposition and subsequent encapsulation and functionalization techniques. Adapted with permission from de Ávila et al. (2016). Copyright 2016 American Chemical Society.

Traditional medical examinations involve the analysis of human tissues using various physical and chemical methods within fields like microbiology, immunology, biochemistry, hematology, and cytology. With the advancement of precision medicine, there is a growing demand for higher levels of accuracy and specificity in these examinations. However, traditional medical examination methods have their limitations. Biosensors, as a method of inspection, can detect biological information within samples. Traditional biosensors often require strict sample environments. In contrast, micro/nanorobots used as sensors exhibit characteristics such as sensitivity and speed, holding significant promise within the field of medicine (Deng et al., 2021; Hegde et al., 2023). One classic biochemical detection technique is the Enzyme-Linked Immunosorbent Assay (ELISA). This method quantifies analytes through enzymatic reactions, followed by chemical colorimetry. ELISA has gained widespread use in the medical field (Aydin, 2015). Wang et al. (2022) developed a micro/nano motor designed for immune probes, enhancing the efficiency of Enzyme-Linked Immunosorbent Assay (ELISA) using Fe3O4@SiO2 core-shell nanorods. These nanorods were further modified with a capture antibody (AB-1) to create a mobile analytical probe based on nanorobots (see Figure 1A). These nanorobots are propelled by a magnetic field and can be guided to target positions through the influence of gradient magnetic fields and rotating magnetic fields. Due to their one-dimensional structure, these robots can rotate and agitate liquids under the influence of a magnetic field, thereby improving the detection efficiency. In an effort to enhance the sensitivity and specificity of virus and protein detection, Mayorga-Martinez et al. (2022) developed a magnetic microrobot (see Figure 1B). This microrobot was modified with anti-SARS coronavirus type 2 spike protein and loaded with pre-concentrated spike protein. Additionally, anti-SARS coronavirus type 2 spike protein coupled with Ag-AuNRs was employed as a marker. This detection platform exhibits heightened sensitivity, reducing the detection limit for severe acute respiratory syndrome coronavirus type 2 by an order of magnitude when compared to traditional detection methods. The utilization of this microrobot platform for virus and protein detection can be applied to similar virus or protein detection scenarios.

Utilizing the microrobot platform, individuals can achieve the detection of toxins. de Ávila et al. (2016) devised a self-propelled micromotor constructed from reduced graphene oxide (RGO) and platinum (Pt). They modified this micromotor with a specialized ricin B aptamer labeled with fluorescein-amidine (FAM) dye (see Figure 1C). Through a visual “on-off” fluorescence reaction and rapid binding to toxins, this system enables quick and sensitive detection across various media. Consequently, trace amounts of ricin can be detected in complex biological samples, and the advancement of this microrobot platform holds significant promise for detecting other biological threats, making it invaluable for biological defense applications. In a separate development, Zhang et al. (2019) introduced an efficient, spore-based fluorescent magnetic robot. They integrated porous natural spores with Fe3O4 particles and coupled them with functionalized carbon dots (CDs), allowing for tracking through fluorescence emission. These microrobots can rapidly detect *Clostridium difficile* toxin, with their detection efficiency confirmed on bacterial culture medium and clinical stool specimens.

### 2.2 Dignosis

Traditional medical examinations primarily operate at a macro level, making it challenging to access deep tissues without causing patient discomfort or trauma, as seen in procedures like endoscopy or biopsy. Additionally, certain imaging examinations, such as those involving radiation or magnetic resonance imaging, can be impacted by metal interference or patient movement. Micro/nanorobots possess remarkable tissue-penetrating capabilities. Furthermore, they can be externally controlled to navigate through the body’s internal environment, thereby enhancing the efficiency and precision of various medical applications (Aziz et al., 2020). These applications include tumor detection, enhanced imaging signal acquisition, pathological biopsy, and more (Tu et al., 2017; Yang, 2020).

Micro/nanorobots have the potential to replace conventional endoscopes in diagnosing intestinal diseases. Traditional colonoscopy, a well-established clinical practice for direct intestinal observation and biopsy, has certain drawbacks. Its larger size relative to microscopic objects can potentially cause discomfort or injury to patients during the procedure. Moreover, there are inherent limitations in observing and operating in certain blind spots within the intestine during examinations (Tapia-Siles et al., 2016; Tumino et al., 2023). In response to these challenges, technological advancements have led to the development of non-invasive endoscopic capsules and capsule endoscopes that can actively navigate within the intestinal tract under magnetic field guidance. However, these solutions also have their limitations (Bianchi et al., 2019). To address these issues, Han et al. (2020) introduced an innovative micro-robotic model for diagnosing intestinal diseases. This micro-robot, designed using principles of bionics, features a two-layer folding mechanism to anchor itself to intestinal tissue. Additionally, it includes a stretching mechanism that allows for axial motion within the intestine, with radio waves serving as the means of propulsion. Robots with this design can move both forward and backward within the intestine while securely attached to the intestinal wall. The safety and effectiveness of this microrobot have been confirmed through *in vitro* experiments conducted in pig intestines.

Micro/nanorobots offer significant advantages in enhancing medical imaging by reaching deep tissues and providing strong penetration capabilities, ultimately leading to clearer images. Photoacoustic imaging is an innovative imaging technique that combines the benefits of optical resolution and acoustic penetration, and it finds a wide range of clinical applications (Attia et al., 2019; Manohar and Gambhir, 2020; Gröhl et al., 2021). Yan et al. (2020) and others devised a microrobot made from a mixture of photoresist and nickel particles. They control the movement of this microrobot using an external magnetic field and utilize photoacoustic imaging for imaging purposes. Their verification experiments demonstrated that this microrobot exhibits high sensitivity in turbid biological tissues. Magnetic imaging is another widely used method for visualizing microrobots (Zhang et al., 2022a). In comparison to other imaging techniques, magnetic imaging offers superior resolution and penetration depth while avoiding the ionizing radiation associated with some imaging methods. During the research and development of microrobots, magnetic materials are incorporated to generate distinct signals from surrounding tissues in a magnetic field. Yan et al. (2017) prepared a spirulina micro robot coated with Fe3O4, offering superparamagnetic properties and enabling it to move in a targeted manner within biological fluids under the influence of an external magnetic field. This microrobot serves as a contrast imaging tool, enhancing imaging performance. Furthermore, it can be biodegradable and exhibits selective cytotoxicity towards cancer cells.

**FIGURE 2:**
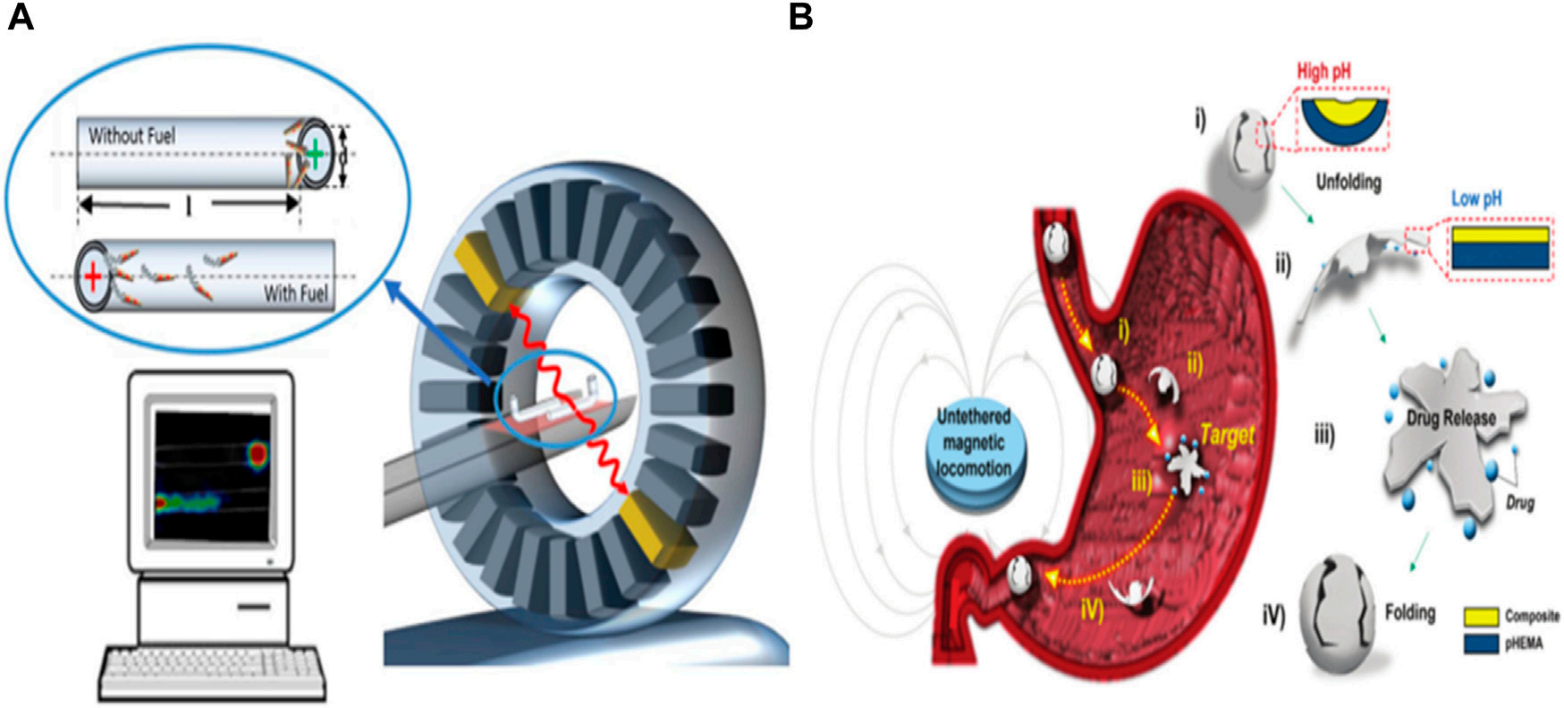
**(A)** PET-CT for tracking tubular Au/PEDOT/Pt micromotors. Adapted with permission from Vilela et al. (2018). Copyright 2018 American Chemical Society. **(B)** Conceptual design of the pH-responsive microrobot for targeted drug delivery, X-ray imaging, and retrieval. Reproduced with permission from Darmawan et al. (2022); published by Royal Society of Chemistry, 2022.

Micro/nanorobots also play a vital role in ionization imaging, significantly enhancing diagnostic capabilities. Ionization imaging is a prevalent method for disease diagnosis in clinical practice, and one such technique is Proton Emission Tomography (PET), which relies on radioactive nuclides. PET is particularly effective at detecting deep tissue distribution of radioactive nuclides. However, it may lack anatomical and morphological details, leading to its frequent combination with CT and MRI in clinical settings (Wållberg and Ståhl, 2013). One drawback of nuclide-based examinations is their high equipment cost, limited real-time imaging capabilities, and lengthy acquisition times. Dahroug et al. (2018) addressed this by developing a microrobot coated with an iodine isotope surface, tracked using PET-CT. Their work confirmed PET-CT’s ability to image microrobot models within deep tissues, demonstrating the technique’s capacity to locate microrobots (see Figure 2A). By incorporating magnetic nanoparticles and a pH-responsive design, a micro robot can be propelled by an external magnetic field while being equipped with an X-ray contrast agent for real-time X-ray imaging (see Figure 2B) (Darmawan et al., 2022). This versatile microrobot, carrying doxorubicin, can facilitate real-time imaging diagnostics and combat tumor cells in gastric cancer.

### 2.3 Targeting drug delivery

**FIGURE 3:**
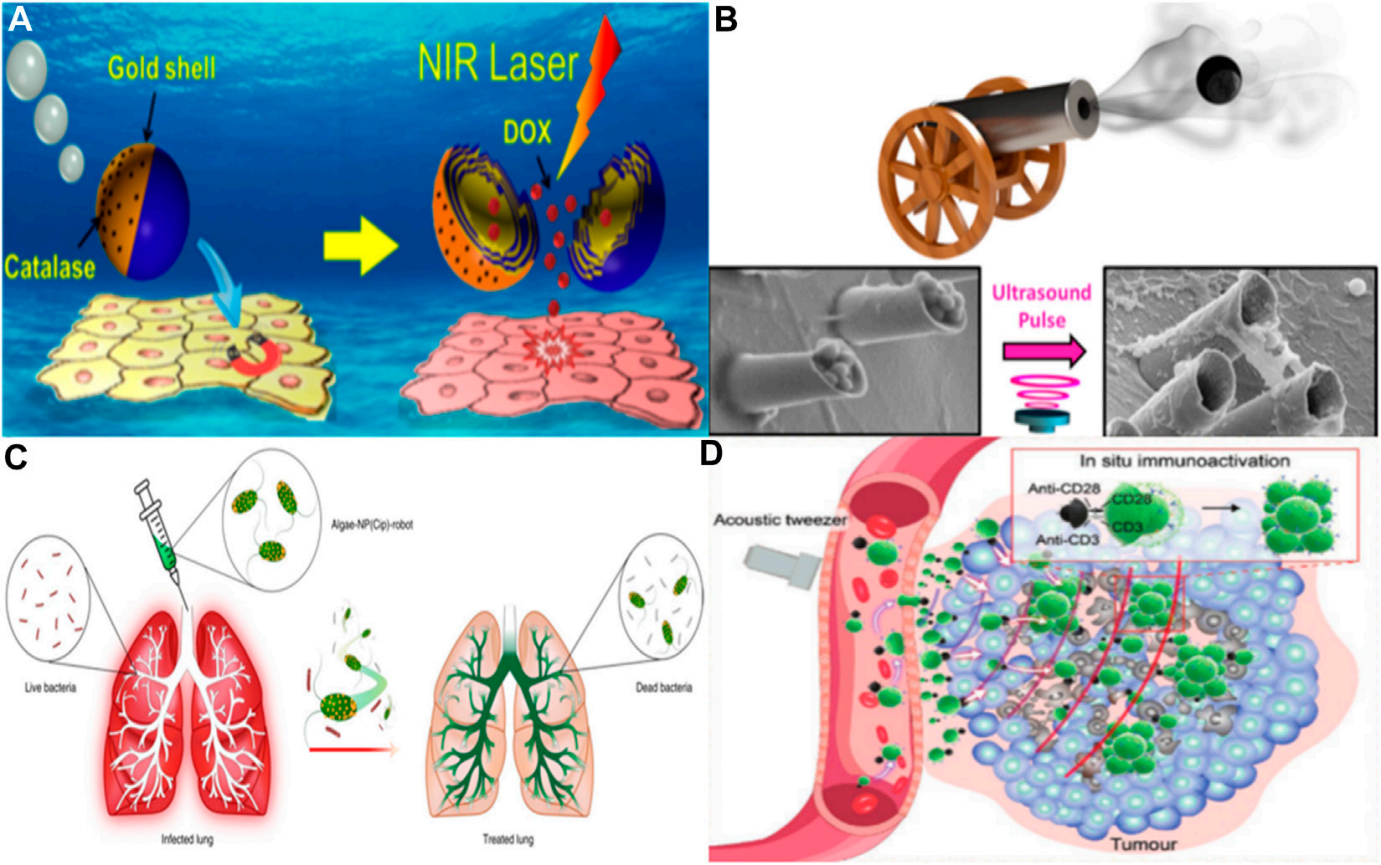
**(A)** Janus capsule engine navigates to the cell layer through an external magnetic field and releases drugs activated by near-infrared light. Adapted with permission from Wu et al. (2014). Copyright 2014 American Chemical Society. **(B)** Acoustic triggered micro gun, which can load and launch nano bullets, is a powerful micro ballistic tool. It can efficiently load and launch a variety of goods, and provide better target location and enhanced tissue penetration performance. Adapted with permission from Soto et al. (2016). Copyright 2016 American Chemical Society. **(C)** Schematic depicting the use of algae-NP-robot for the treatment of a bacterial lung infection. C. *reinhardtii* algae is modified with drug-loaded NPs and then administered *in vivo* for the treatment of P. *aeruginosa* lung infection. Reprinted with the permission from Zhang et al. (2022b). Copyright 2022 Nature Publishing Group. **(D)** Peritumoural-accumulated M-CAR Ts were propelled by acoustic force from tumor-fixed acoustic tweezers to migrate deeply into the deep tumor. Immunomagnetic beads modified with anti-CD3/CD28 *in situ* stimulated the proliferation and activation of CAR T cells, thus achieving a robust antitumor effect. Reproduced with permission from Tang et al. (2023); published by John Wiley and Sons, 2023.

The traditional drug delivery route primarily involves systemic circulation, but it has limitations such as poor tissue permeability and local drug delivery capability, resulting in inefficient drug delivery. However, with the advancement of microrobot technology, these challenges can be overcome. By utilizing microrobots to transport drugs, it becomes possible to target deep tissues for precise drug delivery, leading to increased delivery efficiency and reduced drug dosages (Li et al., 2017a; Li et al., 2017b; Ji et al., 2021). Microrobots can even navigate biofilm structures to deliver drugs, and this drug delivery method has been extensively studied in conditions like inflammation and tumors (Yang et al., 2017; Lin et al., 2021). In the realm of drug delivery, there is a growing desire for microrobots to autonomously navigate to affected areas for precise treatment (Fan et al., 2022). Consequently, the means of propelling these robots is a critical challenge that requires resolution. Early solutions involved the design of micro/nanorobots driven by chemistry, which converts chemical energy into kinetic energy, enabling them to self-propel. Such robots can transport and release various substances, including drugs, nucleic acids, and microorganisms (Wu et al., 2013). Wu et al. (2014) developed a chemically driven polymer-based microrobot that can be securely released upon near-infrared light stimulation. By incorporating biological enzyme catalysis, these robots can self-propel even at lower hydrogen peroxide fuel concentrations, significantly advancing the potential of micro/nanorobots for drug delivery (see Figure 3A).

Beyond chemical propulsion, prevalent methods for actuation encompass magnetic, optical, and even pH-based approaches (Wu et al., 2016; Chen et al., 2018; Yu et al., 2018; Yu et al., 2023). Magnetic propulsion, in particular, stands out as a safer alternative, eschewing the byproducts of chemical propulsion that could potentially disrupt human circulatory and tissue systems. Numerous micro- and nanorobotic systems harnessing magnetic drive have been engineered, showcasing enhanced drug delivery efficiencies both *in vitro* and *in vivo* (Gong et al., 2022). These microrobots are often embedded with magnetic particles or affixed with permanent magnets, allowing for precision control by modulation of magnetic field direction and intensity to realize targeted therapeutic delivery. Mu et al. (2022) devised a hydrogel-based microrobot for drug delivery, utilizing a synthetic lethal drug that, while inherently cytotoxic to normal cells, is guided by genomic markers. Through magnetic manipulation, the microrobot devised by Mu and others can be steered with precision to tumor sites, thereby mitigating adverse effects and augmenting the efficacy of drug delivery. Peng et al. (2017) demonstrated the capabilities of a magnetically-driven nanorobot. They ingeniously incorporated magnetic metallic nickel *in situ* into catalytic platinum nanoparticles, ensuring the synergistic coexistence of nickel and platinum. This integration enables the nanorobot to be driven in dual modes. The controllability of the nanorobot was validated using a human cervical cancer cell model, heralding significant potential for precise drug delivery applications. In targeting the digestive system for drug administration, pH-sensitive mechanisms are often employed to steer robots (Gao et al., 2012; Sattayasamitsathit et al., 2014). Gao et al. (2015) unveiled an acid-powered, zinc-based microrobot capable of efficient propulsion and targeted drug delivery within acidic environments, negating the need for conventional fuel sources. *In vivo* experiments using mice demonstrated that, unlike traditional orally administered drugs that rely on passive diffusion, these micro/nanorobots actively navigate and persist within the gastric walls. This zinc-based microrobot is designed to decompose in acidic conditions without releasing harmful substances, thereby proving non-toxic to human tissues. Further advancing the field, their team proposed a magnesium-based microrobot tailored for treating *Helicobacter pylori* infections by delivering antibiotics. This microrobot possesses a proton depletion feature, potentially reducing the reliance on proton pump inhibitors and minimizing side effects associated with their long-term usage, as compared to traditional passive drug diffusion methods (de Ávila et al., 2017). Soto et al. (2016) introduced acoustic microcannons that harness sound wave energy for propulsion and manipulation of minute payloads. By incorporating a magnetic component, the microcannons can be precisely positioned during the drug release process. These researchers aspire to use acoustic cannons, either singly or in arrays, to transport drugs and vaccines, thus broadening their practical utility. They aim to minimize resistance by optimizing the microcannons’ shape and density, enabling the “nano bullets” to penetrate target tissues efficiently without excessive energy discharge. Such microcannons have the potential for a variety of applications, including drug delivery, tissue penetration, and destruction, actualizing the concept of a “magic bullet” (see Figure 3B).

In the realm of medical applications, micro/nanorobots confront several challenges, including the penetration of biofilm structures and the navigation and drug administration within specialized tissues such as the vitreous body and the lungs. The latter organ, replete with macrophages designed to eliminate foreign entities, poses a particular challenge: evading the human immune system’s clearance mechanisms to achieve targeted drug delivery remains a formidable hurdle for micro/nanorobotic technologies. Zhang et al. (2022b) have engineered a microrobot system tailored for intratracheal drug delivery (see Figure 3C). This system comprises microrobots cloaked in the cell membrane of neutrophils, with a core of algae modified with nanoparticles that ferry therapeutic agents. In contrast to purely synthetic constructs, these biologically hybridized microrobots inherit the intrinsic properties of their biological components. They exhibit facile cultivation, self-propulsion via flagellar motion, and improved distribution and retention within pulmonary tissues. Notably, the flagellar activity has been demonstrated to decrease macrophage-mediated clearance of the microrobots. The presence of the neutrophil cell membrane not only shields the microrobots from the host’s biological milieu but also facilitates targeted pathogen binding and drug delivery. Substantiated through both *in vivo* and *in vitro* studies, the efficacy of this biologically hybrid microrobot system in treating bacterial pneumonia has been confirmed. In recent years, advancements in biomimetics and micro/nanorobotics have given rise to a burgeoning array of bio-integrated microrobot systems (Middelhoek et al., 2022). These systems are designed to harness biological features to diminish the rate of immune clearance by the body and to enhance the efficiency of drug delivery (Li et al., 2016; Yu et al., 2022; Zhang et al., 2023). Li et al. (2023) have pioneered the design of a biomimetic micro-robot, which emulates the claw-like appendages of certain aquatic microorganisms. This novel design offers a significant advancement over traditional synthetic micro-robots. The claw structure enables the robot to anchor to the vascular endothelium in a manner akin to these organisms, rendering it less susceptible to the shearing forces of blood flow. Such an adaptation ensures active retention within the circulatory system. Furthermore, the researchers have ingeniously enveloped the micro-robot with a layer of red blood cell membrane, capitalizing on its role as a natural transport medium. This coating effectively camouflages the robot, mitigating immune detection and clearance. During *in vivo* trials in the rabbit jugular vein, the biomimetic robot exhibited proficient propulsive behavior and demonstrated its potential for active, targeted drug delivery. The blood-brain barrier (BBB) represents a formidable physiological barricade segregating the brain’s milieu from the systemic circulation. While it serves as a protective shield against exogenous substances, potentially detrimental to neural tissue, it concurrently poses a significant challenge for delivering therapeutic agents to the brain (Kadry et al., 2020). Joseph et al. (2017) designed a synthetic vesicle with chemotaxis to cross the blood-brain barrier. This is a fully synthesized organic nanorobot that encapsulates glucose oxidase alone or together with catalase into nanoscale vesicles. These vesicles demonstrate sensitive movement toward regions with higher glucose concentrations, following the external glucose gradient. Moreover, they have exhibited that the chemotactic ability of these nanorobots, coupled with their affinity for the low-density lipoprotein receptor-related protein 1 (LRP-1), enhances their permeability across the BBB by fourfold. Nanorobots are also emerging as instrumental tools in the rapidly advancing domain of cancer immunotherapy. While Chimeric Antigen Receptor T (CAR-T) cell therapy has shown promise in treating hematological malignancies, its efficacy in solid tumors is curtailed by physical barriers and the immune microenvironment (Ma et al., 2019; Li et al., 2021). Addressing this, Tang et al. (2023) have engineered a living micro-robot using CAR-T cells, which are augmented with immune magnetic beads through click conjugation (see in Figure 3D). This construct is magnetically steerable and capable of navigating fluids and circumventing obstacles. It demonstrates the capacity for long-range travel and accumulation in tumor models, showing an impressive ability to penetrate deep into tumor tissues aided by acoustic waves, thereby enhancing the infiltration of exogenous CD8^+^ T cells sixfold. The inclusion of anti-CD3/CD28 immune magnetic beads promotes the activation of the infiltrating CAR-T cells, substantially bolstering their anti-tumor efficacy. In conclusion, nanorobots represent a paradigm shift in enhancing the precision and efficiency of drug delivery, overcoming environmental barriers, and refining targeted therapeutic interventions. It is the collective hope of the research community that these advanced microrobot systems will soon transition into clinically viable tools for healthcare practitioners.

### 2.4 Surgery

As surgical methodologies evolve, particularly with the advent of minimally invasive techniques, there is an escalating demand for reduced trauma and increased precision in reaching surgical targets. Traditional approaches, whether open or minimally invasive, confront limitations in the depth of tissue accessibility and the inability to operate under significantly microscopic conditions. The emergence of micro/nanorobotics in surgical arenas promises to mitigate these challenges. These diminutive robots are designed to navigate and congregate precisely at targeted sites, directed by external stimuli. Their compact form factor enables traversal through capillary networks to locations that are otherwise inaccessible via conventional surgical methods, facilitating interventions at the cellular scale. Such capability not only reduces the scale of surgical incisions but also abbreviates the recuperation period, heralding significant prospects for clinical treatments (Nelson et al., 2010). Micro/nanorobots offer the distinct advantage of operating at considerable distances with robust tissue penetration capabilities. Xi et al. (2013) have pioneered a remotely controllable “surgical knife,” a testament to the innovative strides in this field. This groundbreaking work presents a novel magnetically-guided micro drill designed for minimally invasive surgery. Fabricated from 2D nanomaterials with acutely sharp tips, these microsurgical tools can dynamically alter their orientation by modulating the frequency of an external magnetic field in tandem with the solution’s viscosity. The reported experimental data suggests that these micro drills can execute precise drilling tasks in soft biological tissues and navigate through fluid mediums mirroring the viscosity of blood. Such magnetic-controlled micro drills hold immense promise for minimally invasive medical interventions, including targeted micro drug delivery and the removal of vascular plaques. Further extending the scope of microsurgical innovation, Liu et al. (2022) introduced a micro robot capable of conducting intricate surgical procedures within the confines of microscale blood vessels. This magnetic-driven, soft continuum micro robot is fabricated from a blend of polydimethylsiloxane (PDMS) and neodymium iron boron (NdFeB) particles, allowing for precision navigation and manipulation via magnetic control. Experimental demonstrations validate the soft continuum micro robot’s significant flexibility and particle manipulation prowess, rendering it highly effective for operational tasks within microvascular structures. However, further improvements are required, particularly in terms of the size and control accuracy of soft continuous microrobots. Nguyen et al. (2018) also showcased a micro/nanorobot designed for intravascular surgery. This system comprises a spiral microrobot guided by specially manufactured wires and an enhanced electromagnetic excitation system. Experiments have demonstrated its capacity to generate a maximum magnetic field with nearly zero phase delay across a broad operating frequency range. The system offers precise motion control and cutting force, making it suitable for thrombectomy procedures within the vascular system. In the future, *in vivo* experiments can be conducted to assess the clinical effectiveness of the mechanical thrombectomy device proposed in this study.

**FIGURE 4:**
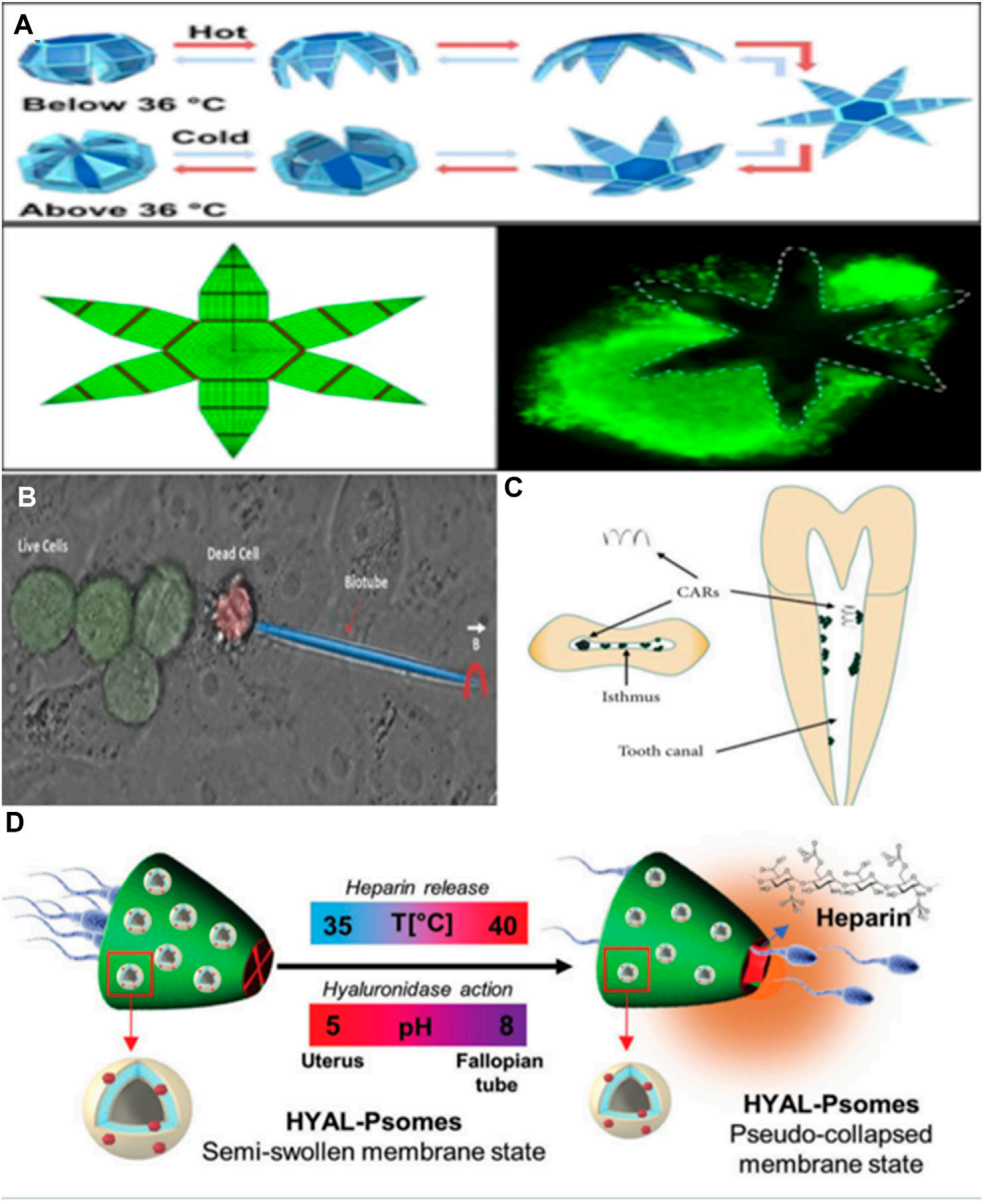
**(A)** A hydrogel robot can perform surgical operations. Adapted with permission from Breger et al. (2015). Copyright 2015 American Chemical Society. **(B)** A double acting micro robot that can create cell incisions. Reproduced with permission from Srivastava et al. (2016); published by John Wiley and Sons, 2016. **(C)** A magnetic microrobot for catalytic biofilm degradation. Reproduced with permission (Cheng et al., 2022). Copyright 2022, Chen Cheng. **(D)** A multifunctional microcomputer robot carrying sperm to realize utilization. Reproduced with permission from Rajabasadi et al. (2022); published by John Wiley and Sons, 2022.

The ability of microrobots to penetrate tissues can be harnessed for tissue and specimen biopsies. With the ongoing advancements in minimally invasive surgery technology, there is a growing need to miniaturize surgical instruments for procedures conducted through natural openings or small incisions. Currently, one of the primary features of minimally invasive surgical instruments is their reliance on connecting wires or cables to transmit energy and signals while being controlled externally. These connecting wires restrict the flexibility of surgical tools and their capacity to access hard-to-reach areas within the body. Gultepe et al. (2013) have pioneered the development of wireless and autonomous microsurgical tools designed for accessing narrow channels inside the body. These microtools can autonomously move and grasp tissues through thermal activation. Constructed from thermally sensitive materials, they can be controlled using external heat sources. The researchers demonstrated the viability of these microtools for *in vivo* pig bile duct biopsies through experiments, offering a novel approach to *in vivo* pig bile duct biopsies. In the fields of robotics and surgery, the ability to grasp and securely hold objects is a crucial task, particularly when it comes to tissue biopsy. Breger et al. (2015) introduced a novel design and preparation method for a self-folding micro fixture that combines thermosensitive hydrogel and hard polymer. By incorporating iron oxide nanoparticles into the porous hydrogel layer, this micro fixture becomes responsive to magnetic fields, enabling remote control. The micro fixture comprises a rigid PPF segment and a soft PNIPAM AAC layer, which automatically closes when the temperature exceeds 36°C. Experimental results demonstrate that this self-folding micro clamp effectively clamps onto cell tissue, and the clamping and release times can be regulated by temperature adjustments (see Figure 4A).On the other hand, Leong et al. (2009) developed an innovative micro fixture with mobility and quality control capabilities, driven by biological signals. Utilizing the principles of thin-film strain drive and self-assembly, this micro fixture was designed, manufactured, and subjected to experimental validation. The research findings reveal that this micro fixture exhibits excellent grasping and handling capabilities, making it suitable for operation in various environments.

One of our primary objectives is the miniaturization of robots to an incredibly small scale, potentially at the cellular or even subcellular level. These miniature robots could perform surgical operations within this dimension, and even be used for tasks like transmitting or harvesting DNA and RNA. Solovev et al. (2012) developed a self-driving nanotube structure through molecular beam epitaxy. He utilized bubbles generated by the decomposition of hydroxide fuel to propel these nanotubes. By depositing a ferromagnetic layer on the nanotube, remote magnetic guidance of nanotools can also be achieved. These nanotube tools possess a small diameter and a unique asymmetric structure, allowing them to generate spiral motion and perform drilling on biomaterials. These micro/nano tools are capable of carrying multiple yeast cells for transportation. However, it’s worth noting that the propellant used is toxic to mammalian cells, limiting its feasibility for applications involving living cells. In another approach, Srivastava et al. (2016) proposed a micro robot with dual functionality for targeted drug delivery and cell surgery. They extracted calcified porous microneedles from Dracaena species with drug-carrying capabilities and coated them with a magnetic layer for cell penetration under the influence of a magnetic field. The carried drugs have the potential to eliminate nearby harmful and malignant cells while also releasing supplementary drugs to enhance the body’s defense against infections. The robot’s ability to penetrate single cells can also serve as an anchoring mechanism for drug release, especially in secondary treatments mediated by other drugs. This robot system offers the potential to significantly reduce treatment side effects, including those associated with chemotherapy (see Figure 4B). Harder et al. (2023) introduced a microrobot constructed from alginate. This microrobot relies on a combination of photothermal drive and temperature sensing to precisely stimulate a single cell. It activates the intracellular thermosensitive ion channels, leading to changes in intracellular calcium content. While preliminary experiments were conducted on human embryonic kidney cells, and a comprehensive evaluation of toxicity and biocompatibility is yet to be performed, the emergence of this robot platform presents a novel approach for studying the thermobiology of cells and mammalian tissues.

With the ongoing development of microrobot technology, its application in surgical procedures has been gradually transitioning into clinical practice. Even though it is still primarily in the experimental stages with animal subjects, the results obtained thus far inspire confidence. Ullrich et al. (2013) introduced a microrobot designed for minimally invasive surgery in the posterior segment of the eye and presented the outcomes of activity experiments conducted in rabbit eyes. These microdevices are injected into the vitreous cavity and can be guided to the lesion site through wireless control via a magnetic field. Surgeons can then manipulate the microrobots for necessary treatments, such as mechanical procedures or targeted drug delivery. The initial application of this technology focuses on drug delivery to lesions near the retina, aiming to reduce the required drug dosage and prolong treatment duration. This approach allows for injection into the eye, manipulation, and subsequent removal of the microrobot, offering a potential adjunctive method for ophthalmic treatments. However, further research is needed to explore the interaction and impact of these microrobots on intraocular tissues. On a related note, Wu et al. (2018) designed micropropellers capable of moving within the eye under external magnetic field control, enabling precise delivery of drugs and therapeutic substances to target areas. These micropropellers feature a super-lubricated surface coating, reducing friction resistance within the vitreous of the eyeball, allowing them to move freely within the eye. Using technologies like optical coherence tomography (OCT), researchers successfully observed the movement and positioning of these micropropellers in pig eyes, providing a new technique for intraocular surgery and treatment.

Microrobots are increasingly finding applications in various surgical fields. Nguyen et al. (2018) introduced the concept and requirements for micro-robot-assisted cholesteatoma surgery. By combining microscopy, imaging technology, and laser ablation tools, residual cholesteatoma cells can be detected and removed. However, a complete robot system for clinical experiments has not yet been fully developed. Cheng et al. (2022) also summarized the progress of microrobots in dentistry. Microrobots can magnify differences that are imperceptible to the naked eye, leading to more precise treatments. They offer unparalleled advantages in procedures like root canal resection, dental pulp treatment, and even tumor treatment (see Figure 4C). Sperm has become a focal point of research in the micro/nanorobot field due to its flagellar structure and innate self-movement capabilities. Sperm’s ability to evade human immune responses and deliver drugs has led to the development of various microrobot systems (Medina-Sánchez et al., 2018). In the field of assisted reproduction, studies have explored the use of microrobots, prepared using single sperm, for treating conditions such as oligospermia and azoospermia (Magdanz et al., 2017). However, the functions achievable with only a single sperm cell are limited. Subsequently, Rajabasadi et al. (2022) developed a microrobot carrier capable of carrying multiple sperm, constructed from various intelligent materials (see Figure 4D). This microrobot can achieve *in situ* sperm capacitation through localized heparin release and degrade the cumulus complex surrounding the oocyte, thereby enhancing the probability of fertilization.

## 3 Conclusion and future perspective

This paper serves as a comprehensive summary of the remarkable achievements in the application of micro/nanorobots in the field of medicine, particularly in the domains of biosensors, image diagnosis, targeted drug delivery, and minimally invasive surgery. Micro/nano robots offer advantages that traditional treatment methods cannot match, including their ability to reach deep tissues, minimize trauma, and enhance treatment efficiency. Presently, research in this field is still in its infancy, and there remain significant challenges to overcome on the path toward clinical transformation. One primary challenge is ensuring the biosafety of these micro/nano robots. Given their need to navigate deep tissues or even aggregate, it is imperative that these robots exhibit non-toxic and degradable characteristics. Additionally, concerns arise regarding the generation of chemical waste during robot operations, which may potentially harm the human body. While some biodegradable micro/nano robots have been successfully developed, precise control over their degradation environment and timeline remains essential. Moreover, these biodegradable robots primarily undergo degradation in PBS solutions or animal models, leaving their parameters and functions in a human environment largely uncharted. A second significant challenge revolves around the control of these robots and the aspiration to orchestrate their collective movement. Future advancements driven by fields like artificial intelligence may allow for the autonomous movement of robot clusters, even facilitating coordination between individual robots. Such developments hold tremendous promise, especially in complex treatment domains like large-scale medication and tumor immune environment therapy. The third major challenge involves the reduction of material and production costs to enable mass production of these robots. This endeavor intersects with materials science and 3D printing technologies. Researchers aim to explore a broader range of biological and synthetic materials while seeking more efficient and cost-effective fabrication methods. The ultimate goal is to establish a safe and widely accessible micro/nano robot system, ensuring that more individuals can benefit from precise and efficient medical treatments. In conclusion, the journey toward realizing the full potential of micro/nanorobots in the treatment of human diseases is long and multifaceted. Numerous details await exploration, including ethical considerations. It is anticipated that research into the micro/nano robot platform will experience rapid growth in the coming decade. As long as safety and controllability conditions are met, micro/nano robots hold the potential to revolutionize the field of medical treatment.

## References

[B1] Attia A. B. E. Balasundaram G. Moothanchery M. Dinish U. S. Bi R. Ntziachristos V. (2019). A review of clinical photoacoustic imaging: current and future trends. Photoacoustics 16, 100144. 10.1016/j.pacs.2019.100144 31871888 PMC6911900

[B2] Aydin S. (2015). A short history, principles, and types of ELISA, and our laboratory experience with peptide/protein analyses using ELISA. Peptides 72, 4–15. 10.1016/j.peptides.2015.04.012 25908411

[B3] Aziz A. Pane S. Iacovacci V. Koukourakis N. Czarske J. Menciassi A. (2020). Medical imaging of microrobots: toward *in vivo* applications. ACS Nano 14 (9), 10865–10893. 10.1021/acsnano.0c05530 32869971

[B4] Bianchi F. Masaracchia A. Shojaei Barjuei E. Menciassi A. Arezzo A. Koulaouzidis A. (2019). Localization strategies for robotic endoscopic capsules: a review. Expert Rev. Med. Devices 16 (5), 381–403. 10.1080/17434440.2019.1608182 31056968

[B5] Breger J. C. Yoon C. Xiao R. Kwag H. R. Wang M. O. Fisher J. P. (2015). Self-folding thermo-magnetically responsive soft microgrippers. ACS Appl. Mater Interfaces 7 (5), 3398–3405. 10.1021/am508621s 25594664 PMC4326779

[B6] Chen X. Z. Jang B. Ahmed D. Hu C. De Marco C. Hoop M. (2018). Small-scale machines driven by external power sources. Adv. Mater 30 (15), e1705061. 10.1002/adma.201705061 29443430

[B7] Cheng C. Yinan X. Zongxin X. Lei S. Yanan X. Yanli Y. (2022). Robotic and microrobotic tools for dental therapy. J. Healthc. Eng. 2022, 1–12. 10.1155/2022/3265462 PMC8881140 35222881

[B8] Dahroug B. Tamadazte B. Weber S. Tavernier L. Andreff N. (2018). Review on otological robotic systems: toward microrobot-assisted cholesteatoma surgery. IEEE Rev. Biomed. Eng. 11, 125–142. 10.1109/RBME.2018.2810605 29994589

[B9] Darmawan B. A. Gong D. Park H. Jeong S. Go G. Kim S. (2022). Magnetically controlled reversible shape-morphing microrobots with real-time X-ray imaging for stomach cancer applications. J. Mater Chem. B 10 (23), 4509–4518. 10.1039/d2tb00760f 35616358

[B10] de Ávila B. E. F. Angsantikul P. Li J. Angel Lopez-Ramirez M. Ramírez-Herrera D. E. Thamphiwatana S. (2017). Micromotor-enabled active drug delivery for *in vivo* treatment of stomach infection. Nat. Commun. 8 (1), 272. 10.1038/s41467-017-00309-w 28814725 PMC5559609

[B11] de Ávila B. E. F. Lopez-Ramirez M. A. Báez D. F. Jodra A. Singh V. V. Kaufmann K. (2016). Aptamer-modified graphene-based catalytic micromotors: off–on fluorescent detection of ricin. ACS Sens. 1, 217–221. 10.1021/acssensors.5b00300

[B12] Deng J. Zhao S. Liu Y. Liu C. Sun J. (2021). Nanosensors for diagnosis of infectious diseases. ACS Appl. Bio Mater 4 (5), 3863–3879. 10.1021/acsabm.0c01247 35006812

[B13] Fan X. Jiang Y. Li M. Zhang Y. Tian C. Mao L. (2022). Scale-reconfigurable miniature ferrofluidic robots for negotiating sharply variable spaces. Sci. Adv. 8 (37), eabq1677. 10.1126/sciadv.abq1677 36112686 PMC9481141

[B14] Gao W. Dong R. Thamphiwatana S. Li J. Gao W. Zhang L. (2015). Artificial micromotors in the mouse's stomach: a step toward *in vivo* use of synthetic motors. ACS Nano 9 (1), 117–123. 10.1021/nn507097k 25549040 PMC4310033

[B15] Gao W. Uygun A. Wang J. (2012). Hydrogen-bubble-propelled zinc-based microrockets in strongly acidic media. J. Am. Chem. Soc. 134 (2), 897–900. 10.1021/ja210874s 22188367

[B16] Gong D. Celi N. Zhang D. Cai J. (2022). Magnetic biohybrid microrobot multimers based on *chlorella* cells for enhanced targeted drug delivery. ACS Appl. Mater Interfaces 14 (5), 6320–6330. 10.1021/acsami.1c16859 35020358

[B17] Gröhl J. Schellenberg M. Dreher K. Maier-Hein L. (2021). Deep learning for biomedical photoacoustic imaging: a review. Photoacoustics 22, 100241. 10.1016/j.pacs.2021.100241 33717977 PMC7932894

[B18] Gultepe E. Randhawa J. S. Kadam S. Yamanaka S. Selaru F. M. Shin E. J. (2013). Biopsy with thermally-responsive untethered microtools. Adv. Mater 25 (4), 514–519. 10.1002/adma.201203348 23047708 PMC3832625

[B19] Han D. Yan G. Wang Z. Jiang P. Liu D. Zhao K. (2020). The modelling, analysis, and experimental validation of a novel micro-robot for diagnosis of intestinal diseases. Micromachines (Basel) 11 (10), 896. 10.3390/mi11100896 32992512 PMC7601751

[B20] Harder P. İyisan N. Wang C. Kohler F. Neb I. Lahm H. (2023). A laser-driven microrobot for thermal stimulation of single cells. Adv. Healthc. Mater 12, e2300904. 10.1002/adhm.202300904 37229536 PMC11468149

[B21] Hegde C. Su J. Tan J. M. R. He K. Chen X. Magdassi S. (2023). Sensing in soft robotics. ACS Nano 17 (16), 15277–15307. 10.1021/acsnano.3c04089 37530475 PMC10448757

[B22] Ji F. Li T. Yu S. Wu Z. Zhang L. (2021). Propulsion gait analysis and fluidic trapping of swinging flexible nanomotors. ACS Nano 15 (3), 5118–5128. 10.1021/acsnano.0c10269 33687190

[B23] Joseph A. Contini C. Cecchin D. Nyberg S. Ruiz-Perez L. Gaitzsch J. (2017). Chemotactic synthetic vesicles: design and applications in blood-brain barrier crossing. Sci. Adv. 3 (8), e1700362. 10.1126/sciadv.1700362 28782037 PMC5540238

[B24] Kadry H. Noorani B. Cucullo L. (2020). A blood-brain barrier overview on structure, function, impairment, and biomarkers of integrity. Fluids Barriers CNS 17 (1), 69. 10.1186/s12987-020-00230-3 33208141 PMC7672931

[B25] Leong T. G. Randall C. L. Benson B. R. Bassik N. Stern G. M. Gracias D. H. (2009). Tetherless thermobiochemically actuated microgrippers. Proc. Natl. Acad. Sci. U. S. A. 106 (3), 703–708. 10.1073/pnas.0807698106 19139411 PMC2630075

[B26] Li H. Wang Z. Ogunnaike E. A. Wu Q. Chen G. Hu Q. (2021). Scattered seeding of CAR T cells in solid tumors augments anticancer efficacy. Natl. Sci. Rev. 9 (3), nwab172. 10.1093/nsr/nwab172 35265340 PMC8900686

[B27] Li T. Chang X. Wu Z. Li J. Shao G. Deng X. (2017b). Autonomous collision-free navigation of microvehicles in complex and dynamically changing environments. ACS Nano 11 (9), 9268–9275. 10.1021/acsnano.7b04525 28803481

[B28] Li T. Li J. Morozov K. I. Wu Z. Xu T. Rozen I. (2017a). Highly efficient freestyle magnetic nanoswimmer. Nano Lett. 17 (8), 5092–5098. 10.1021/acs.nanolett.7b02383 28677387

[B29] Li T. Li J. Zhang H. Chang X. Song W. Hu Y. (2016). Magnetically propelled fish-like nanoswimmers. Small 12 (44), 6098–6105. 10.1002/smll.201601846 27600373

[B30] Li T. Yu S. Sun B. Li Y. Wang X. Pan Y. (2023). Bioinspired claw-engaged and biolubricated swimming microrobots creating active retention in blood vessels. Sci. Adv. 9 (18), eadg4501. 10.1126/sciadv.adg4501 37146139 PMC10162671

[B31] Lin R. Yu W. Chen X. Gao H. (2021). Self-propelled micro/nanomotors for tumor targeting delivery and therapy. Adv. Healthc. Mater 10 (1), e2001212. 10.1002/adhm.202001212 32975892

[B32] Liu D. Liu X. Chen Z. Zuo Z. Tang X. Huang Q. (2022). Magnetically driven soft continuum microrobot for intravascular operations in microscale. Cyborg Bionic Syst. 2022, 9850832. 10.34133/2022/9850832 36285316 PMC9494713

[B33] Ma L. Dichwalkar T. Chang J. Y. H. Cossette B. Garafola D. Zhang A. Q. (2019). Enhanced CAR-T cell activity against solid tumors by vaccine boosting through the chimeric receptor. Science 365 (6449), 162–168. 10.1126/science.aav8692 31296767 PMC6800571

[B34] Magdanz V. Medina-Sánchez M. Schwarz L. Xu H. Elgeti J. Schmidt O. G. (2017). Spermatozoa as functional components of robotic microswimmers. Adv. Mater 29 (24), 201606301. 10.1002/adma.201606301 28323360

[B35] Manohar S. Gambhir S. S. (2020). Clinical photoacoustic imaging. Photoacoustics 19, 100196. 10.1016/j.pacs.2020.100196 32612928 PMC7317224

[B36] Mayorga-Martinez C. C. Vyskočil J. Novotný F. Bednar P. Ruzek D. Alduhaishe O. (2022). Collective behavior of magnetic microrobots through immuno-sandwich assay: on-the-fly COVID-19 sensing. Appl. Mater Today 26, 101337. 10.1016/j.apmt.2021.101337 35018299 PMC8739527

[B37] Medina-Sánchez M. Xu H. Schmidt O. G. (2018). Micro- and nano-motors: the new generation of drug carriers. Ther. Deliv. 9 (4), 303–316. 10.4155/tde-2017-0113 29540126

[B38] Middelhoek KINA Magdanz V. Abelmann L. Khalil I. S. M. (2022). Drug-Loaded IRONSperm clusters: modeling, wireless actuation, and ultrasound imaging. Biomed. Mater 17 (6), 8. 10.1088/1748-605X/ac8b4b 35985314

[B39] Mohammadinejad R. Karimi S. Iravani S. Varma R. S. (2016). Plant-derived nanostructures: types and applications. Green Chem. 18, 20–52. 10.1039/c5gc01403d

[B40] Mu H. Liu C. Zhang Q. Meng H. Yu S. Zeng K. (2022). Magnetic-driven hydrogel microrobots selectively enhance synthetic lethality in MTAP-deleted osteosarcoma. Front. Bioeng. Biotechnol. 10, 911455. 10.3389/fbioe.2022.911455 35875497 PMC9299081

[B41] Nelson B. J. Kaliakatsos I. K. Abbott J. J. (2010). Microrobots for minimally invasive medicine. Annu. Rev. Biomed. Eng. 12, 55–85. 10.1146/annurev-bioeng-010510-103409 20415589

[B42] Nguyen K. T. Go G. Choi E. Kang B. Park J. O. Kim C. S. (2018). A guide-wired helical microrobot for mechanical thrombectomy: a feasibility study. Annu. Int. Conf. IEEE Eng. Med. Biol. Soc. 2018, 1494–1497. 10.1109/EMBC.2018.8512455 30440675

[B43] Nguyen K. T. Kim S. J. Min H. K. Hoang M. C. Go G. Kang B. (2021). Guide-wired helical microrobot for percutaneous revascularization in chronic total occlusion in-vivo validation. IEEE Trans. Biomed. Eng. 68 (8), 2490–2498. 10.1109/TBME.2020.3046513 33351745

[B44] Peng F. Tu Y. Men Y. van Hest J. C. Wilson D. A. (2017). Supramolecular adaptive nanomotors with magnetotaxis behavior. Adv. Mater 29 (6), 201604996. 10.1002/adma.201604996 27891683

[B45] Rajabasadi F. Moreno S. Fichna K. Aziz A. Appelhans D. Schmidt O. G. (2022). Multifunctional 4D-printed sperm-hybrid microcarriers for assisted reproduction. Adv. Mater 34 (50), e2204257. 10.1002/adma.202204257 36189842

[B46] Sattayasamitsathit S. Kou H. Gao W. Thavarajah W. Kaufmann K. Zhang L. (2014). Fully loaded micromotors for combinatorial delivery and autonomous release of cargoes. Small 10 (14), 2830–2833. 10.1002/smll.201303646 24706367 PMC4107182

[B47] Solovev A. A. Xi W. Gracias D. H. Harazim S. M. Deneke C. Sanchez S. (2012). Self-propelled nanotools. ACS Nano 6 (2), 1751–1756. 10.1021/nn204762w 22233271

[B48] Soto F. Martin A. Ibsen S. Vaidyanathan M. Garcia-Gradilla V. Levin Y. (2016). Acoustic microcannons: toward advanced microballistics. ACS Nano 10 (1), 1522–1528. 10.1021/acsnano.5b07080 26691444

[B49] Soto F. Wang J. Ahmed R. Demirci U. (2020). Medical micro/nanorobots in precision medicine. Adv. Sci. (Weinh) 7 (21), 2002203. 10.1002/advs.202002203 33173743 PMC7610261

[B50] Srivastava S. K. Medina-Sánchez M. Koch B. Schmidt O. G. (2016). Medibots: dual-action biogenic microdaggers for single-cell surgery and drug release. Adv. Mater 28 (5), 832–837. 10.1002/adma.201504327 26619085

[B51] Tang X. Yang Y. Zheng M. Yin T. Huang G. Lai Z. (2023). Magnetic-acoustic sequentially actuated CAR T cell microrobots for precision navigation and *in situ* antitumor immunoactivation. Adv. Mater 35 (18), e2211509. 10.1002/adma.202211509 36807373

[B52] Tapia-Siles S. C. Coleman S. Cuschieri A. (2016). Current state of micro-robots/devices as substitutes for screening colonoscopy: assessment based on technology readiness levels. Surg. Endosc. 30 (2), 404–413. 10.1007/s00464-015-4263-1 26092000

[B53] Tu Y. Peng F. Wilson D. A. (2017). Motion manipulation of micro- and nanomotors. Adv. Mater 29 (39). 10.1002/adma.201701970 28841755

[B54] Tumino E. Visaggi P. Bolognesi V. Ceccarelli L. Lambiase C. Coda S. (2023). Robotic colonoscopy and beyond: insights into modern lower gastrointestinal endoscopy. Diagn. (Basel) 13 (14), 2452. 10.3390/diagnostics13142452 PMC10378494 37510196

[B55] Ullrich F. Bergeles C. Pokki J. Ergeneman O. Erni S. Chatzipirpiridis G. (2013). Mobility experiments with microrobots for minimally invasive intraocular surgery. Invest. Ophthalmol. Vis. Sci. 54 (4), 2853–2863. 10.1167/iovs.13-11825 23518764

[B56] Vilela D. Cossío U. Parmar J. Martínez-Villacorta A. M. Gómez-Vallejo V. Llop J. (2018). Medical imaging for the tracking of micromotors. ACS Nano 12 (2), 1220–1227. 10.1021/acsnano.7b07220 29361216

[B57] Wållberg H. Ståhl S. (2013). Design and evaluation of radiolabeled tracers for tumor imaging. Biotechnol. Appl. Biochem. 60 (4), 365–383. 10.1002/bab.1111 24033592

[B58] Wang Y. Liu X. Chen C. Chen Y. Li Y. Ye H. (2022). Magnetic nanorobots as maneuverable immunoassay probes for automated and efficient enzyme linked immunosorbent assay. ACS Nano 16 (1), 180–191. 10.1021/acsnano.1c05267 35015504

[B59] Wu Y. Lin X. Wu Z. Möhwald H. He Q. (2014). Self-propelled polymer multilayer Janus capsules for effective drug delivery and light-triggered release. ACS Appl. Mater Interfaces 6 (13), 10476–10481. 10.1021/am502458h 24909305

[B60] Wu Z. Si T. Gao W. Lin X. Wang J. He Q. (2016). Superfast near-infrared light-driven polymer multilayer rockets. Small 12 (5), 577–582. 10.1002/smll.201502605 26690728

[B61] Wu Z. Troll J. Jeong H. H. Wei Q. Stang M. Ziemssen F. (2018). A swarm of slippery micropropellers penetrates the vitreous body of the eye. Sci. Adv. 4 (11), eaat4388. 10.1126/sciadv.aat4388 30406201 PMC6214640

[B62] Wu Z. Wu Y. He W. Lin X. Sun J. He Q. (2013). Self-propelled polymer-based multilayer nanorockets for transportation and drug release. Angew. Chem. Int. Ed. Engl. 52 (27), 7000–7003. 10.1002/anie.201301643 23703837

[B63] Xi W. Solovev A. A. Ananth A. N. Gracias D. H. Sanchez S. Schmidt O. G. (2013). Rolled-up magnetic microdrillers: towards remotely controlled minimally invasive surgery. Nanoscale 5 (4), 1294–1297. 10.1039/c2nr32798h 23154823 PMC4151060

[B64] Xie H. Sun M. Fan X. Lin Z. Chen W. Wang L. (2019). Reconfigurable magnetic microrobot swarm: multimode transformation, locomotion, and manipulation. Sci. Robot. 4 (28), eaav8006. 10.1126/scirobotics.aav8006 33137748

[B65] Yan X. Zhou Q. Vincent M. Deng Y. Yu J. Xu J. (2017). Multifunctional biohybrid magnetite microrobots for imaging-guided therapy. Sci. Robot. 2 (12), eaaq1155. 10.1126/scirobotics.aaq1155 33157904

[B66] Yan Y. Jing W. Mehrmohammadi M. (2020). Photoacoustic imaging to track magnetic-manipulated micro-robots in deep tissue. Sensors (Basel) 20 (10), 2816. 10.3390/s20102816 32429159 PMC7287980

[B67] Yang J. (2020). Janus microdimer swimming in an oscillating magnetic field. R. Soc. Open Sci. 7 (12), 200378. 10.1098/rsos.200378 33489250 PMC7813250

[B68] Yang R. Wei T. Goldberg H. Wang W. Cullion K. Kohane D. S. (2017). Getting drugs across biological barriers. Adv. Mater 29 (37). 10.1002/adma.201606596 PMC5683089 28752600

[B69] Yu H. Tang W. Mu G. Wang H. Chang X. Dong H. (2018). Micro-/Nanorobots propelled by oscillating magnetic fields. Micromachines (Basel) 9 (11), 540. 10.3390/mi9110540 30715039 PMC6266240

[B70] Yu S. Li T. Ji F. Zhao S. Liu K. Zhang Z. (2022). Trimer-like microrobots with multimodal locomotion and reconfigurable capabilities. Mater. Today Adv. 14, 100231. 10.1016/j.mtadv.2022.100231

[B71] Yu S. Sun Z. Zhang Z. Sun H. Liu L. Wang W. (2021). Magnetic microdimer as mobile meter for measuring plasma glucose and lipids. Front. Bioeng. Biotechnol. 9, 779632. 10.3389/fbioe.2021.779632 34900967 PMC8660689

[B72] Yu Z. Li L. Mou F. Yu S. Zhang D. Yang M. (2023). Swarming magnetic photonic-crystal microrobots with on-the-fly visual pH detection and self-regulated drug delivery. InfoMat 5, e12264. 10.1002/inf2.12464

[B73] Zhang F. Zhuang J. Li Z. Gong H. de Ávila B. E. Duan Y. (2022b). Nanoparticle-modified microrobots for *in vivo* antibiotic delivery to treat acute bacterial pneumonia. Nat. Mater 21 (11), 1324–1332. 10.1038/s41563-022-01360-9 36138145 PMC9633541

[B74] Zhang W. Deng Y. Zhao J. Zhang T. Zhang X. Song W. (2023). Amoeba-inspired magnetic venom microrobots. Small 19 (23), e2207360. 10.1002/smll.202207360 36869412

[B75] Zhang Y. Zhang L. Yang L. Vong C. I. Chan K. F. Wu W. K. K. (2019). Real-time tracking of fluorescent magnetic spore-based microrobots for remote detection of *C. diff* toxins. Sci. Adv. 5 (1), eaau9650. 10.1126/sciadv.aau9650 30746470 PMC6357761

[B76] Zhang Z. Wang H. Yang H. Song W. Dai L. Yu S. (2022a). Magnetic microswarm for MRI contrast enhancer. Chem. Asian J. 17 (17), e202200561. 10.1002/asia.202200561 35791774

[B77] Zhao S. Sun D. Zhang J. Lu H. Wang Y. Xiong R. (2022). Actuation and biomedical development of micro-/nanorobots-A review. Mater. Today Nano 18, 100223. 10.1016/j.mtnano.2022.100223

